# Genetic integrity of the human Y chromosome exposed to groundwater arsenic

**DOI:** 10.1186/1755-8794-3-35

**Published:** 2010-08-06

**Authors:** Safdar Ali, Sher Ali

**Affiliations:** 1 Molecular Genetics Laboratory, National Institute of Immunology, Aruna Asaf Ali Marg, New Delhi-110067, India

## Abstract

**Background:**

Arsenic is a known human carcinogen reported to cause chromosomal deletions and genetic anomalies in cultured cells. The vast human population inhabiting the Ganges delta in West Bengal, India and Bangladesh is exposed to critical levels of arsenic present in the groundwater. The genetic and physiological mechanism of arsenic toxicity in the human body is yet to be fully established. In addition, lack of animal models has made work on this line even more challenging.

**Methods:**

Human male blood samples were collected with their informed consent from 5 districts in West Bengal having groundwater arsenic level more than 50 μg/L. Isolation of genomic DNA and preparation of metaphase chromosomes was done using standard protocols. End point PCR was performed for established sequence tagged sites to ascertain the status of recombination events. Single nucleotide variants of candidate genes and amplicons were carried out using appropriate restriction enzymes. The copy number of DYZ1 array per haploid genome was calculated using real time PCR and its chromosomal localization was done by fluorescence in-situ hybridization (FISH).

**Results:**

We studied effects of arsenic exposure on the human Y chromosome in males from different areas of West Bengal focusing on known recombination events (P5-P1 proximal; P5-P1 distal; gr/gr; TSPY-TSPY, b1/b3 and b2/b3), single nucleotide variants (SNVs) of a few candidate Y-linked genes (DAZ, TTY4, BPY2, GOLGA2LY) and the amplicons of AZFc region. Also, possible chromosomal reorganization of DYZ1 repeat arrays was analyzed. Barring a few microdeletions, no major changes were detected in blood DNA samples. SNV analysis showed a difference in some alleles. Similarly, DYZ1 arrays signals detected by FISH were found to be affected in some males.

**Conclusions:**

Our Y chromosome analysis suggests that the same is protected from the effects of arsenic by some unknown mechanisms maintaining its structural and functional integrities. Thus, arsenic effects on the human body seem to be different compared to that on the cultured cells.

## Background

Several heavy metals are present in the environment all over the world in amounts alarmingly unsafe for the human population of which chromium and arsenic are good examples. These metals affect human systems in various ways but their possible genetic consequences remain unknown. In the context of arsenic, Ganges delta in West Bengal, India and Bangladesh, both area- and population wise are the worlds most affected regions. In Bangladesh, over 60% of villages are at the risk from arsenic exposure [1].

Arsenic in the environment exists naturally in two forms; as arsenite (trivalent As^3+^) or arsenate (pentavalent As^5+^). Humans are exposed to arsenic by ingestion of contaminated water, food and drugs or inhalation from burning of arsenic contaminated coal. Inhalation is also contributed by semiconductor and glass manufacturing sites. Arsenic is present in small to trace amounts in rocks, sediments and all natural water resources which includes rivers, sea water and groundwater. In the absence of treatment process, high levels of arsenic become a major health hazards. The World Health Organization (WHO) recommends less than 10 μg/L arsenic in drinking water and its maximum permissible limit is 50 μg/L [2]. Our present understanding of the metal demands the limit to be set at 10 μg/L but the lack of adequate testing facilities at such low concentrations in countries with this problem makes them adhere to a high permissible limit. The sensitivity of the scenario may be judged by the fact that at consumption of a liter of water per day with 50 μg/L arsenic, 13 per thousand individuals may die due to liver, lung, kidney or bladder cancer [3]. The risk is only reduced to about 37 per 10000 individuals at a level of 10 μg/L which is the lowest of the enacted guidelines across the world [4]. Besides, lesser exposed males are apparently more prone to developing skin lesions as compared to females with far greater exposure. Interestingly both sexes were maximally affected at the same age group of 35-44 years [5].

Arsenite despite being an established human carcinogen, its mechanism of carcinogenesis and genetic effects remain unclear. What is known is that it induces chromosomal aberrations in both human and rodent cell lines and the cells of exposed humans [6-9]. Subsequently, these genetic abnormalities become cause of cancer [10] though their random nature remains to be explained. In addition, its role as a tumor promoter [11] has been suggested without any direct evidence. Another possibility includes its action as a co-mutagen by interfering with DNA repair mechanism, enhancing the effect of mutagens like UV and MNU (N-methyl-N-nitrosourea) [12]. The greatest challenge in understanding arsenic carcinogenicity and its role *in-vivo *has been the absence of animal models since it fails to replicate its effect in rodents [13]. In addition the complexities seem to be increasing from risk of erectile dysfunction in exposed males [14] to its high levels in milk of lactating mothers [15]. Also, estrogen sensitive targets may be responsible for the differential affect in males and females [16]. Most affected regions in India are the 9 districts in West Bengal where the recorded groundwater arsenic level is more than 50 μg/L to which over 40 million people are exposed [17]. We collected human blood samples from these districts and analyzed them for anomalies, if any, focusing on their Y chromosomes.

## Methods

### Collection of blood samples and genomic DNA isolation

Blood samples (10 ml) were collected with informed consent from 98 males from different areas of West Bengal strictly in accordance with the Guidelines of Institutes Ethical and Bio-safety Committee. The regions were selected for having ground water arsenic of more than 50 μg/L as reported earlier [17]. Present study includes samples from 5 districts which include Kolkata, Mednipur, Murshidabad, Maldah and 24 Paragnas (S). The samples in the age group of 7 to 62 years were short listed by confirming that they were consuming ground water as such, without any treatment and were exposed to arsenic for a minimum period of 7 years. From these, 4 individuals (2k10, 2k28, 2k66 and MC7) had skin lesions on faces or hands due to arsenic exposure. Two persons (2k11 and 2k29) had been operated for prostate enlargement. Also, routine blood analysis for cell counts and hemoglobin level was done and only the ones found to be normal were included in the study. In addition blood was collected from 80 males residing in New Delhi without any arsenic exposure and used as controls. Genomic DNA isolation was done from blood using standard protocols [18].

### Sequence-tagged site PCR amplification

STS spanning all the known regions of Y chromosome showing recombination deletions were amplified using end point PCR. These included P5-proximal P1, P5- distal P1, gr/gr, b1/b3 and b2/b3 deletion. Screening was done for deletion of entire AZFa or AZFc region. Recombination events known to occur in AZFa due to the presence of provirus A and B sequences were checked. The AZFb region was analyzed for its intactness using STS markers.

### End point PCR analysis

The reactions in 20 μl volume were carried out using Go Taq polymerase and 5× reaction buffer (Promega, Madison, USA), 200 μM dNTPs and 100 ng of template DNA. The reaction was conducted for 30 cycles, each involving denaturation at 95°C for 1 minute, annealing at 60°C for 1 minute and extension at 72°C for 1 minute besides initial denaturation at 95°C for 5 minutes and final extension at 72°C for 10 minutes. The amplified products were resolved on appropriate agarose gels. β-actin and SRY primers were used as positive controls [19].

### Single Nucleotide Variants (SNV) analysis

For the analysis of SNVs initial PCR amplification was carried out as above in 50 μl reaction mixture. After subsequent confirmation of amplification, PCR product was purified by adding 5 μl of 3 M sodium acetate and 150 μl of absolute ethanol which was then incubated at -70°C for 2 hours. Thereafter, it was pelleted (13 k rpm for 20 minutes) and washed with 70% ethanol before dissolving and putting up for digestion with appropriate restriction enzymes.

### Real time PCR analysis of DYZ1 region

Genomic DNA from different samples was used as template for analysis of number of DYZ1 arrays using Real Time PCR. *Power *SYBR^® ^green (Part No. 4367659) from Applied Biosystems (ABI, USA). Reactions were carried out on Sequence Detection System 7500 (ABI, USA). Ten fold serial dilutions of the cloned DYZ1 plasmid was made starting with 30 crore copies and used for standard curve preparation. The genomic DNA was used in 3 different concentrations 2 ng, 1 ng and 0.5 ng and subsequently copies were calculated per genome for each sample. All the reactions were carried out in triplicates. All the standard curves used had a slope value of 3.3-3.5 and R^2 ^value of >0.99.

### Fluorescence in situ hybridization (FISH)

Approximately, 300 μl of freshly collected blood from both set of samples was cultured in PB Max karyotyping medium (GIBCO) and chromosome preparation was done using standard protocols [20]. A 3.4 Kb clone of the DYZ1 was labeled with biotin-dUTP using Nick Translation Kit from Vysis (IL, USA) and used for FISH following standard protocols [20]. The images were analyzed by Applied Imaging Systems Cytovision software version 3.92.

## Results

### Overall STS analysis for recombination events and chromosome intactness

Samples were checked for the presence of STSs encompassing recombination events and intactness of the azoospermia factor (AZF) regions.

#### AZFc region

**Figure 1:**
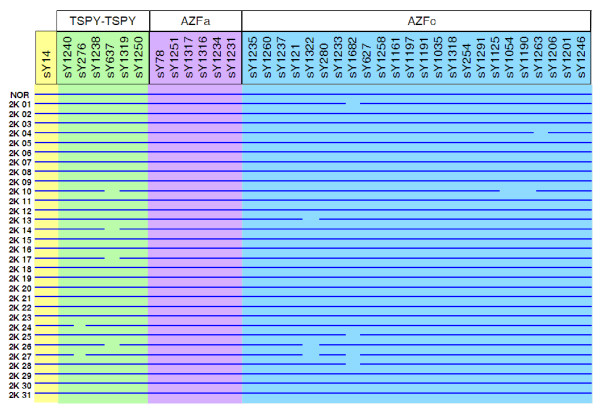
**Screening of STSs across the Y chromosome**. Diagrammatic representation summarizing STS analysis in the representative samples for screening of recombinations in the MSY region of the Y chromosome. We used 37 STSs to screen for different recombination deletions including P5-Proximal P1, P5-Distal P1, gr/gr, b1/b3, b2/b3, TSPY-TSPY besides checking for the presence of AZFa region. sY14 located in SRY gene was used as positive control. The sample IDs are given on the left side while the STSs analyzed are on top. Continuous blue line is indicative of intactness of the STSs screened while the interrupting orange bars reflect absence of the same.

The AZFc region was analyzed for its intactness and occurrence of different recombination events (P5-P1 proximal; P5-P1 distal; gr/gr; b1/b3 and b2/b3) as per the details given in table 1[21-30]. Besides, the TSPY-TSPY recombination was also accounted for (sY1240, sY1250 positive and sY276, sY1238, sY637, sY1319 all negative). None of the samples showed any of these described recombination events. However, there were random microdeletions in some samples. Results on STS mapping of representative samples are shown in figure 1.

**Table 1:** STSs screening for recombinations in the AZFc region of human Y chromosome

	sY 1235	sY 1260	sY 1237	sY 121	sY 1322	sY 280	sY 1233	sY 1682	sY 627	sY 142	sY 1258	sY 1161	sY 1197	sY 1191	sY 1035	sY 1318	sY 254	sY 1291	sY 1125	sY 1054	sY 1190	sY 1263	sY 1206	sY 1201	sY 1246
P5-P1 Proximal	+	+	-	-	-	-	-	-	-	-	-	-	-	-	+	+	+	-	+	+	+	+	+	+	+

P5-P1 Distal	+	+	-	-	-	-	-	-	-	-	-	-	-	-	-	-	-	-	+	+	+	+	-	+	+

AZFc	+	+	+	+	+	+	+	+	+	+	+	+	+	-	-	-	-	-	+	-	-	-	-	+	+

gr/gr	+	+	+	+	+	+	+	+	+	+	+	+	+	+	+	+	+	-	+	+	+	+	+	+	+

b1/b3	+	+	+	+	+	+	+	+	+	+	+	-	-	-	+	+	+	-	+	+	+	+	+	+	+

b2/b3	+	+	+	+	+	+	+	+	+	+	+	+	+	-	+	+	+	+	+	+	+	+	+	+	+

#### AZFa region

**Figure 2:**
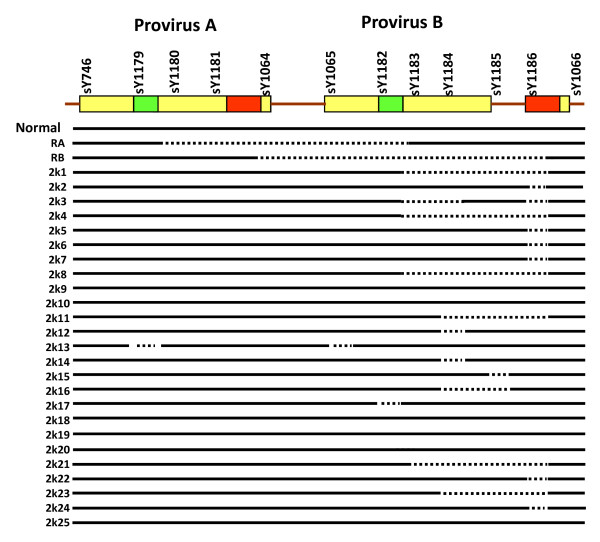
**STS analysis of AZFa region**. An illustration summarizing the result in representative samples for screening of recombination due to HERV provirus sequences in the AZFa region. The green and red bars are the location of homologous sequences responsible for recombination. Intact line indicates presence of STSs while dotted line reflects the corresponding deletion. Normal represents person without any recombination event happening; RA represents deletion pattern expected due to recombination of sequences of the green bar while RB represents pattern due to that of red bar. The STSs are given on top while sample IDs are given on left.

The presence of AZFa region was ascertained by standard STS mapping involving six STSs sY78, sY1251, sY1317, sY1316, sY1234 and sY1231. The absence of sY1317, sY1316, and sY1234 has been taken to be indicative of AZFa deletion. We did not find absence of any of these STSs instead all of them were intact (Figure 1). Further, the region having HERV provirus sequences was checked for homologous recombination following standard markers [27] and the results are given in Figure 2. No sample had the characteristic provirus A or B mediated recombination. However, several microdeletions mostly confined to the provirus B region were detected.

#### AZFb region

**Figure 3:**
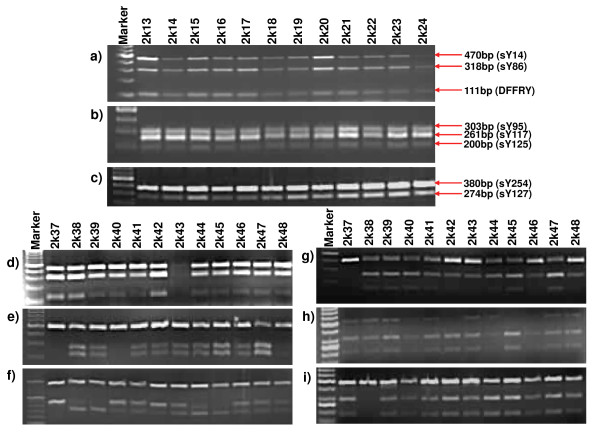
**Multiplex PCR for intactness of AZFb region and SNV analysis in the amplicons and genes**. **a-c **shows representative gel pictures of multiples PCR used for checking the intactness of AZFb region. The sample IDs are shown on top while the amplicon size along with the corresponding STSs is given on right. None of the samples showed any deletion in this region for the STSs screened. **d-g **are the results of representative samples for g, g1, g2 and g3 SFVs respectively. **h-i **represents SNV results for selective samples for TTY4 and BPY2, respectively. For fragment length, restriction enzymes and other details refer to table 2.

The AZFb region was analyzed by multiplex PCR using standard protocol [26]. It involved the screening of 10 STSs including sY86, DFFRY, DDX3Y, sY95, sY117, sY125, sY127, sY254, sY255 and RBMY STSs (Accession no. G73375). All the samples were found to be positive for these STSs (see figure 3a-c) indicating the intactness of AZFb region.

### SNV/SFV analysis

After the recombination analysis, we ascertained the intactness of gene copies and amplicons by single nucleotide variants (SNVs) in the AZFc region. We analyzed 7SNVs in DAZ gene, one each in BPY2, TTY4 and GOLGA2LY genes and 7 located in different amplicons (b2, b3, b4, g1, g2, g3 and Gr)

#### DAZ SNVs

The samples were analyzed for reported SNVs in DAZ gene which included DAZ SNV I to VI [31] and sY581 [32]. The amplicons were studied by end point PCR amplification followed by digestion by corresponding restriction enzymes (table 2). The DAZ deletions were ascertained following standard method [28] that showed intactness of the copies of DAZ gene. One sample 2k44 showed deletion in the DAZ4 gene (DAZ del. haplotype 4).

**Table 2:** The details of SNVs analyzed in the study

Target	SNV	Primers	Enzyme	Fragments	Allele	Present in copies
DAZ Genes SNVs	I	SA770	*Fsp I*	709	A	1,2,3
		SA771		398+311	B	4

	II (sY586)	SA446	*Mbo I*	182	A	1
		SA447		122+60	B	2,3,4

	III	SA772	*Taq I*	301	A	2
		SA773		184+117	B	1,3,4

	IV	SA774	*Alu I*	630	A	2
		SA775		398+262	B	1,3,4

	V (sY587)	SA444	*Dra I*	195+49	A	3,4
		SA445		122+73+49	B	1,2

	VI	SA776	*Afl III*	431	A	1,2,3
		SA777		248+183	B	4

	sY581	SA442	*Sau3a*	189+63	A	1,4
		SA443		130+63+59	B	2,3

Blue Amplicons	b2_AZFc_SFV	SA1237	*MnlI*	653	A	b1, b3, b4
		SA1238		452+197	B	b2

	b3_AZFc_SFV	SA1239	*XmaJI*	510	A	b1, b2, b4
		SA1240		261+249	B	b3

	b4_AZFc_SFV	SA1241	*HphI*	630	A	b1, b2, b3
		SA1242		347+282	B	b4

Green Amplicons	g1_AZFc_SFV	SA1243	*Alw261*	500	A	g2, g3
		SA1244		272+224	B	g1

	g2_AZFc_SFV	SA1245	*NmuCI*	274+180+38	A	g2
		SA1246		274+137+38+28	B	g1, g3

	g3_AZFc_SFV	SA1247	*Alw261*	400	A	g3
		SA1248		251+163	B	g1, g2

Gr Amplicons	g_AZFc_SFV	SA1249	*Eco1051*	440	A	Gr1
		SA1250		311+129	B	Gr2

GOLGA2LY Genes	GOLY/I	SA768	*HhaI*	531	A	1 copy
		SA769		289+242	B	1 copy

BPY2 Genes	BPY2/I	SA766	*EcoRV*	470	A	2 copies
		SA767		289+181	B	I copy

TTY4 Genes	TTY4/I	SA764	*HaeIII*	541	A	1 copy
		SA765		323+218	B	2 copies

#### Amplicons

**Figure 4:**
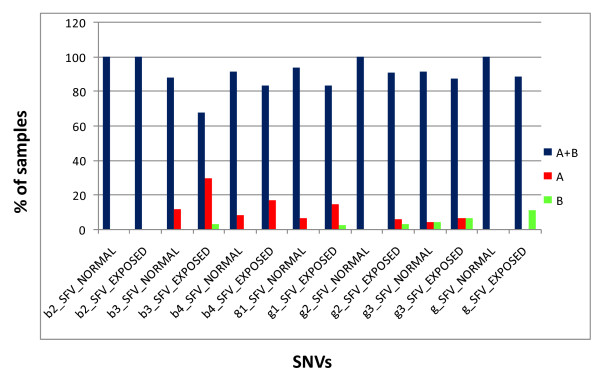
**Result of SNV studies across exposed and normal samples**. Comparative analysis of the results of SNVs present in the amplicons of AZFc region between the unexposed and exposed samples. While b2_SFV has both the alleles present in all the samples (unexposed and exposed) g3_SFv shows a slightly deviating presence of alleles across the samples. The differences seem most profound in the b3_SFV between the two sample sets while in the rest of SFVs show somewhat less variations.

Further, we checked the SNVs in the blue, green and Gr amplicons in the AZFc region following standard protocol [33] to establish their correlations with the normal functioning of the Y chromosome. Representative gel pictures for the same are shown in figure 3d-g. The details of expected fragment pattern are given in table 2 and results summarized in table 3 and figure 4.

**Table 3:** Comparative analysis of SNVs located in the AZFc amplicons between the exposed and unexposed males.

SNV	PROFILE	% OF EXPOSED MALES	% OF UNEXPOSED MALES
b2_AZFc_SFV	A+B	100	100
	
	A	0.0	0.0
	
	B	0.0	0.0

b3_AZFc_SFV	A+B	67.6	88.2
	
	B	29.4	11.8
	
	A	2.9	0.0

b4_AZFc_SFV	A+B	83.3	91.7
	
	A	16.7	8.3
	
	B	0.0	0.0

g1_AZFc_SFV	A+B	83.3	93.8
	
	A	14.6	6.2
	
	B	2.1	0.0

g2_AZFc_SFV	A+B	91.2	100
	
	A	5.9	0.0
	
	B	2.9	0.0

g3_AZFc_SFV	A+B	87.5	91.6
	
	A	6.25	4.2
	
	B	6.25	4.2

g_AZFc_SFV	A+B	88.9	100
	
	A	0.0	0.0
	
	B	11.1	0.0

#### Other AZFc genes

We analyzed the SNV variants of BPY2, TTY4 and GOLGA2LY genes on the Y chromosome. Few samples showed allelic variations (Figure 3h-i) which have been summarized in table 4.

**Table 4:** Results summary of SNVs analyzed in the genes in AZFc region with one sample (2k44) showed DAZ4 deletion haplotype.

Gene	Allele A only	Allele B only
TTY4	-	2k27, 2k28, 2k34, 2k42, 2k45

BPY2	2k12, 2k27, 2k28, 2k35, 2k38	1k4

GOLGA2LY	2k39, 2k40, 1k3, 1k4	2k44, 1k1, 1k8

DAZ SNV I	2K10, 2K18, 2K23, 2K44	-

DAZ SNV III	2K16, 2K17	2K46, 2K47

DAZ SNV VI	2K44	-

### DYZ1 array

The DYZ1 repeats on the human Y chromosome have long been contemplated for their transcriptional status and possible involvement in the chromosome stability [34,35]. We studied its overall intactness and copy number variation in the exposed males.

#### Intactness by end point PCR

**Figure 5:**
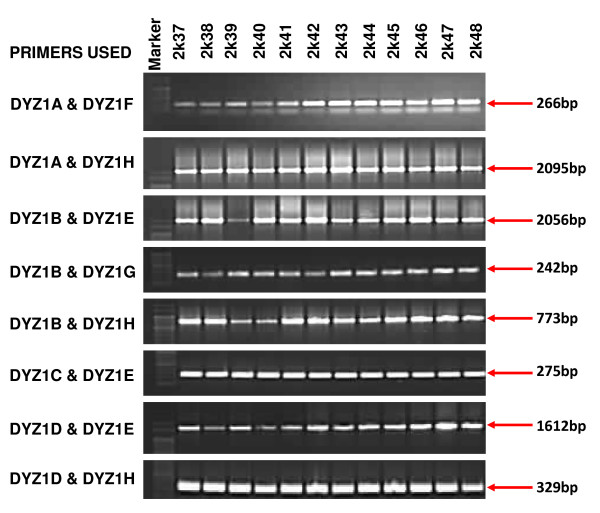
**Analysis of DYZ1 region**. Gel pictures representing analysis of DYZ1 repeat array by end point PCR analysis. Ten different combinations of 8 primers were used for the same (for details refer to tables 4 and 5) and the array was found to be intact this approach in all the samples analyzed.

For studying DYZ1 intactness, we designed 4 sets of primers spanning the entire 3.4 kb array. These primers were then used in 10 different combinations for end point PCR amplification. The details of the primers used, their locations, different combinations and expected amplicons are shown in tables 5 and 6. As per this analysis all the samples (normal and exposed) showed an intact DYZ1 array. The representative pictures related to this analysis are shown in figure 5.

**Table 5:** The primers designed for end point PCR analysis of DYZ1 repeat region.

Primer	Sequence	Location	Orientation	Tm(°C)
DYZ1A	TTTCCTTTCGCTTGCATTCCAT	25-47	5'-3'	65

DYZ1B	TTTTGAGTCCGTTCCATAACAC	1347-1367	5'-3'	64

DYZ1C	GAGTCCATTCACTTCCAGAACA	3128-3149	5'-3'	63

DYZ1D	CCATGCCATTTTATTGCGTTGC	1791-1821	5'-3'	63

DYZ1E	GACTGGAAAGGCTGGGTGTCGA	3380-3402	3'-5'	63

DYZ1F	TGAAATGGACTGGAAAGGAATG	268-290	3'-5'	64

DYZ1G	TGGAATGGACTGCAATAGAAAG	1566-1588	3'-5'	64

DYZ1H	TGGAATGGACTCGAACAGAGTG	2097-2119	3'-5'	64

**Table 6:** The primer combinations from table 3 used for PCR of DYZ1 array and expected amplicons

Sl no	Combination	Amplicon (bp)
1	DYZ1A & DYZ1E	3378

2	DYZ1A & DYZ1F	266

3	DYZ1A & DYZ1G	1564

4	DYZ1A & DYZ1H	2095

5	DYZ1B & DYZ1E	2056

6	DYZ1B & DYZ1G	242

7	DYZ1B & DYZ1H	773

8	DYZ1C & DYZ1E	275

9	DYZ1 D & DYZ1E	1612

10	DYZ1 D & DYZ1H	329

#### Assessment of Copy number variation of DYZ1 by real time PCR

**Figure 6:**
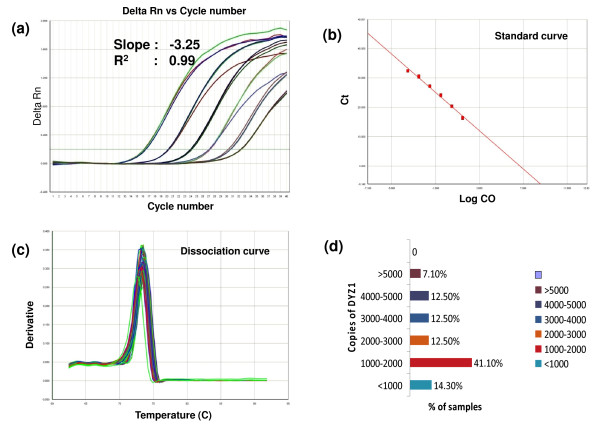
**Copy number assessment of DYZ1 per genome**. **a **represents the amplification plot while **b **and **c **show the corresponding standard plot and dissociation curve respectively. **d **shows the distribution of copy number variation of DYZ1 arrays across the exposed samples based on real time analysis. As clearly evident, 41% of samples have between 1000-2000 copies while 14% samples had less than thousand copies. Contrastingly, all the unexposed samples from Delhi had copies ranging from 3800 to 4200.

We checked number of DYZ1 copies per genome on real time PCR using SYBR green chemistry. Cloned DYZ1 array was used to prepare standard curve by ten-fold serial dilutions starting from 30 crore to 300 copies. The copies per genome in samples were subsequently extrapolated from the standard curve. A representative standard curve along with its amplification plots and dissociation curve has been shown in figure 6a-c. The samples from arsenic exposed areas showed a very high degree of copy number variation ranging from 672 (sample 2k48) to 8576 (sample 2k21). Variation in the unexposed samples was found to be within a lower 3910 to 4200 range. The distribution of variations in copy number across the samples has been summarized in figure 6d.

#### FISH analysis

**Figure 7:**
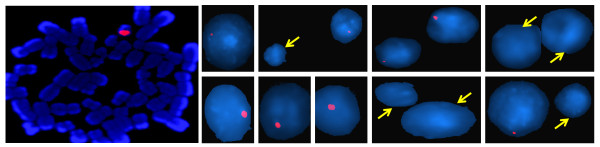
**FISH analysis of DYZ1 array**. The metaphase and interphase nuclei stained with DAPI showing DYZ1 probe signal. Note the variation in intensities across nuclei and absence of signals in some which has been highlighted by arrows.

We used the 3.4 kb clone of DYZ1 array as FISH probe for its chromosomal localization. The signals showed two significant aspects. First, a consistent variation was found in the signal intensity amongst nuclei of the same individual. Secondly, in 19 exposed samples about (20-25%) cells showed no signal. The remaining individuals had signals in >98% cells. To rule out experimental error, a positive control (individual already tested for consistent signals) was used with the same probe preparation. The experiment was replicated several times and each time around 400 nuclei was screened. Representative captured images of one of the samples are shown in figure 7 highlighting these observations. All the cells in the normal control samples showed consistent DYZ1 signals. Also, to ascertain the presence of Y chromosome, WCP-Y spectrum green (Cat no. 32-122024) was purchased from VYSIS (Illinois, USA) and used as reported earlier [36]. It showed signals in >96% nuclei of all the individuals. Analysis of nuclei with specific probes for different regions of DYZ1 array is underway.

## Discussion

Arsenic is a known source of human carcinogen though the mechanism of its carcinogenesis is still not clear. The metabolism of arsenic involves methylation steps subsequent to which monomethylarsanous acid (MMA) and dimethylarsinous acid (DMA) are produced in mammals [37]. It was long believed that the methylation steps constitute a yet to be elucidated detoxification process. However, the fact that bacteria and fungi can successfully survive arsenic exposure and demethylate the arsenic suggests that the methylation is an effect of arsenic [38,39]. Further, mice have been found to be highly resistant to arsenic toxicity. Though C3 H and CD1 mice show increased liver tumor incidence exposed to drinking water arsenic [40-43], it has yet to be confirmed. This is because subsequent experiments on C3 H mice by Ahlborn et al 2009 [44] reported a significant reduction in tumor occurrence (0%) and also that the arsenic exposure given was far exceeding the limit (85 ppm) what humans were exposed to. Assessment of arsenic on the human Y chromosome was undertaken to uncover its possible effect.

In our study, none of the exposed samples showed any of the established recombination/deletion events of the human Y chromosome except a few random microdeletions. In our earlier reports in males exposed to natural background radiation showed a similar pattern but the occurrence of genetical changes was at a higher frequency [36]. In our study, deletion was restricted to provirus B region (see figure 2) which is due to the presence of short stretches of homologous sequences [36]. These changes are attributed to the effect of arsenic exposure since the unexposed samples lack such deletions.

Arsenic has been known to cause deletions in cell lines but in the absence of animal models, it is impossible to undertake an *in-vivo *study. Even in the cell lines, several anomalies were reported but their mechanism remains to be elucidated. On what basis arsenic chooses genetic targets are yet to be uncovered and any specific preference for a particular chromosome or sequence is being probed at. There are multiple reports establishing the involvement of arsenic in sister chromatid exchange (SCE) and chromosomal aberrations in cultured cells [45-47]. None of these or subsequent reports have focussed on the Y chromosome. However, it may be noted that the human Y chromosome contains sizable part of palindromic and repeat sequences which makes it susceptible to chromosomal rearrangements, deletions and recombination. In view of the ability of arsenic to induce such aberrations, Y chromosome provides an ideal setting for such study. We further plan to study the integrity of Y chromosome in cell lines when exposed to arsenic.

The possible role of Y haplogroups also needs to be accounted for prior to any conclusions. In present study, samples were collected from northern India belonging to Indo-European origins which predominantly contains R haplogroup with variations in the DYS marker series [48,49]. Due to the expected uniform distribution of this haplogroup in control and affected sampling regions, we believe deviations between the two sample-groups cannot be significantly attributed to haplogroups. We hypothesize that arsenic in the human body behaves distinctly different as compared to that in established cell lines. Perhaps human body is lot more efficient to counteract the adverse effects of arsenic compared to an individual cell or established cell lines.

SNV analysis showed only one sample with DAZ 4 del haplotype which seems to be a random occurrence and the results of ampliconic SNVs seem to be biased. Of the 7 SNVs in the amplicons of AZFc region, only one located in b2 amplicon showed identical pattern of SNV in normal and exposed samples. The other 6 SNVs showed variation across the sample sets with maximum one in b3 amplicon (figure 4 and table 3).

Most startling aspect of our study was the data on the DYZ1 repeat array. Though the PCR analysis by primers along its length presented a normal picture in all the samples, real time and FISH data were found to be more revealing. The number of copies present in samples varied from 672 (2k48) to 8576 (2k21) with an astounding 55% samples having less than 2000 copies per genome (Figure 6d). The unexposed samples on the other hand also showed arrays copy number variation but in much smaller range. Further, variation in signal intensity within the cell population of same individual after FISH and absence of signal in ~20% cells in 19 samples seems to be an indicative of the arsenic effects on DYZ1 array. The selective absence of signals from certain percentage of cells might be indicative of arsenic induced aneuploidy. In this context, chromosomal analysis at the sequence and mapping level is required to resolve this issue. Interestingly, the samples which were showing arsenic skin lesions did not show any apparent bias towards the aberrations whatsoever. This highlights our sparingly inconsequent understanding of arsenic in the human body.

## Conclusions

We conclude that arsenic is indeed affecting the human Y chromosome at a low level and apparently repeat regions are more prone as evident from our DYZ1 study. Though present study is surely an indicative of some arsenic manifestations in the body, a large scale screening of the exposed samples at the genetic level is required to substantiate the effects of arsenic exposure on the human system. The potential role of repeat regions being involved in arsenic induced carcinogenesis can further be investigated. Absence of a reliable animal model would continue to dodge the efforts on this line but sustained efforts would surely yield the mysteries behind action of arsenic on human body.

## Abbreviations

AZF: Azoospermia Factor; BPY: Basic charge, Y-linked; DAZ: Deleted in Azoospermia; DDX3Y: DEAD (Asp-Glu-Ala-Asp) box polypeptide 3, Y-linked; DMA: Dimethylarsinous Acid; FISH: Fluorescence In Situ Hybridization; GOLGA2LY: Golgi- antigene 2-like Y; MMA: Monomethylarsanous Acid; MNU: N-methyl-N-nitrosourea; RBMY: RNA-binding motif gene on Y chromosome; SCE: Sister Chromatid Exchange; SFV: Sequence Family Variants; SNV: Single Nucleotide Variant; STS: Sequence Tagged Site; TSPY: Testis-Specific Protein Y encoded; TTY4: Testes Transcript Y 4; WHO: World Health Organization.

## Competing interests

The authors declare that they have no competing interests.

## Authors' contributions

Safdar A: carried out the studies and drafted the manuscript. Sher A: conceived of the study, participated in its design and coordination and helped to draft the manuscript. Both authors read and approved the final manuscript.

## Pre-publication history

The pre-publication history for this paper can be accessed here:

http://www.biomedcentral.com/1755-8794/3/35/prepub
